# Surgical site infections after glioblastoma surgery: boon or bane?

**DOI:** 10.1007/s00432-023-05528-x

**Published:** 2024-01-26

**Authors:** Harold F. Hounchonou, Genis Bajgora, Majid Esmaeilzadeh, Christian Hartmann, Joachim K. Krauss

**Affiliations:** 1 Department of Neurosurgery, Hannover Medical School, Carl-Neuberg-Straße 1, 30625 Hannover, Germany; 2 Department of Neuropathology, Hannover Medical School, Carl-Neuberg-Straße 1, 30625 Hannover, Germany

**Keywords:** Surgical site infections, Wound infections, Glioblastoma, Brain cancer

## Abstract

**Background:**

Surgical site infections (SSIs) are among the most common postoperative complications. Glioblastoma multiforme is the most frequent malignant brain tumor with a dismal prognosis despite combined treatment. The effect of SSIs on the course of glioblastoma patients has not been fully clarified since available data are limited and partially contradictory. The aim of this study is to investigate the impact of SSIs on the course of patients with glioblastoma.

**Methods:**

The medical records of all patients undergoing surgery for glioblastoma between 2010 and 2020 in our institution were scanned and those with surgical site infections after glioblastoma resection were identified and compared to an age-matched control group. Overall survival and progression-free survival were the primary endpoints followed by the number of hospitalizations and the length of stay in hospital.

**Results:**

Out of 305 patients undergoing surgery for glioblastoma, 38 patients with postoperative surgical site infection after resection were identified and 15 (5 men and 10 women aged between 9 and 72) were included in this study. 23 patients were excluded. The control group consisted of 30 age-matched patients without SSI (18 men and 12 women). There were no significant differences in median overall survival. Progression-free survival was higher in the SSI group. The number of hospitalizations and the length of stay were significantly higher in the SSI group.

**Conclusion:**

Our data suggest that SSIs might reduce early recurrences without affecting overall survival. Furthermore, they might decrease health-related quality of life by doubling the total length of hospital stay.

**Supplementary Information:**

The online version contains supplementary material available at 10.1007/s00432-023-05528-x.

## Introduction

Glioblastomas are malignant fast-growing brain tumors with a dismal prognosis and limited therapeutic options (Schaff and Mellinghoff 2023). Although the global incidence is relatively low with approximately 5–10 per 100,000 person-years, they represent almost 50% of all gliomas (Dolecek et al. 2012; Rock et al. 2012; Thakkar et al. 2014). Established therapy concepts include surgical resection and radio-chemotherapy (Wen et al. 2020). The clinical outcome in glioblastoma patients remains poor and median survival after maximal therapy is about 15–18 months (Gilbert et al. 2013; Stupp et al. 2005). Furthermore, patients suffer from a deterioration in health-related quality of life (HRQOL) related to neurological symptoms, neurocognitive dysfunction and side effects of adjuvant therapy (Flechl et al. 2017; Sagberg et al. 2016; Ståhl et al. 2022).

Surgical site infections (SSIs) are defined as cutaneous, subcutaneous or deep tissues infections occurring within 30 days (or 1 year if an implant was used; e.g., titanium plates for bone flap fixation) after a surgical procedure at the operation site (Mangram et al. 1999; Owens and Stoessel 2008). They are amongst the most common postoperative complications and occur in up to 20% of all surgeries in Europe depending on the surgical procedure (Leaper et al. 2004; Mangram et al. 1999; Owens and Stoessel 2008). In brain tumors, SSIs have been reported to occur in 4–8% of the surgeries (Scheer et al. 2023; Uzuka et al. 2017). For a long time, it has been hypothesized that bacterial SSIs might positively affect survival time in cancer patients (Kazim et al. 2021; Nauts 1989). In the late nineteenth and early twentieth century, the orthopedic surgeon and cancer researcher William B. Coley even inoculated bacterial organisms (Coley's toxins) into more than 1000 patients with inoperable cancer and reported an “anti-cancer effect” of bacterial infections (Coley 1898, 1991; McCarthy 2006). Subsequently, several investigators have reported and confirmed an anti-tumor effect of bacterial SSIs in different types of tumors (Huh et al. 2019; Nauts et al. 1953; Schantz et al. 1980). Later on, Coley’s method became controversial and was abandoned (McCarthy 2006). While few articles have investigated the potential effect of SSIs on survival in glioblastoma providing contradictory results (Alexiou et al. 2015; Bohman et al. 2009; Bowles & Perkins 1999; Chen et al. 2017; De Bonis et al. 2011; Salle et al. 2021; Walker & Pamphlett 1999), the impact on the length of hospital stay has not been evaluated yet.

Here, we investigate the potential effect of SSIs on overall survival and progression-free survival time and evaluate the impact of SSIs on the number of hospitalizations and the total length of hospital stay (LOS) considering these two parameters as a major key factor impacting HRQOL.

## Methods

### Study design

A registry of all patients undergoing surgery for high-grade glioma in our institution between 2010 and 2020 was established. We retrospectively scanned the medical records of all patients in order to detect those with SSI after glioblastoma surgery. After identifying all patients with SSIs, we established a control group with randomly selected age-matched patients undergoing surgery for glioblastoma in the same period but without experiencing SSIs.

Patients’ data were recorded including demographic findings (age, gender and BMI), dates and types of surgery, survival time and date of death, type of infection, detected pathogens, prescribed antibiotics, postoperative adjuvant therapy protocols, and hospitalization dates. In addition to the age, gender and BMI, further variables including the Karnofsky Performance Scale (KPS), comorbidities, adjuvant therapy, time to adjuvant therapy, extent of tumor resection were included in the analysis. The KPS was determined based on the clinical presentation at the time of diagnosis.

Primary outcomes of this study were overall survival time followed by progression-free survival time. Overall survival was calculated as time from primary surgery to death. Progression-free survival was calculated as the time between the primary surgery and the date of the MRI showing a recurrence or the date of death. As additional endpoints, we also analyzed the frequency of glioblastoma related hospitalizations and LOS.

### Statistical analysis

Patient’s age, BMI and the time to adjuvant therapy are expressed as mean ± SEM. KPS is expressed as median ± SEM. Differences between both groups were analyzed using Student’s t-test for age and time to adjuvant therapy, and the Mann–Whitney test for BMI and KPS. The extent of resection, adjuvant therapy, comorbidities and gender were compared using Fisher´s exact test. The overall survival time and the progression-free survival time were assessed using the Kaplan–Meier-method, expressed as median ± SEM. Differences were analyzed with the Log-rank test and the Gehan-Breslow-Wilcoxon test. The number of hospitalizations and the LOS are expressed as mean ± SEM and the group comparison was done with Student´s t-test.

Statistical analyses and graphs were performed with GraphPad Prism (GraphPad Prism 9.3.0 (345) Macintosh Version by Software MacKiev © 1994–2021 GraphPad Software, LLC).

Statistical significance is defined as *P* < 0.05.

## Results

### Cohort description and patient characteristics

**Fig. 1:**
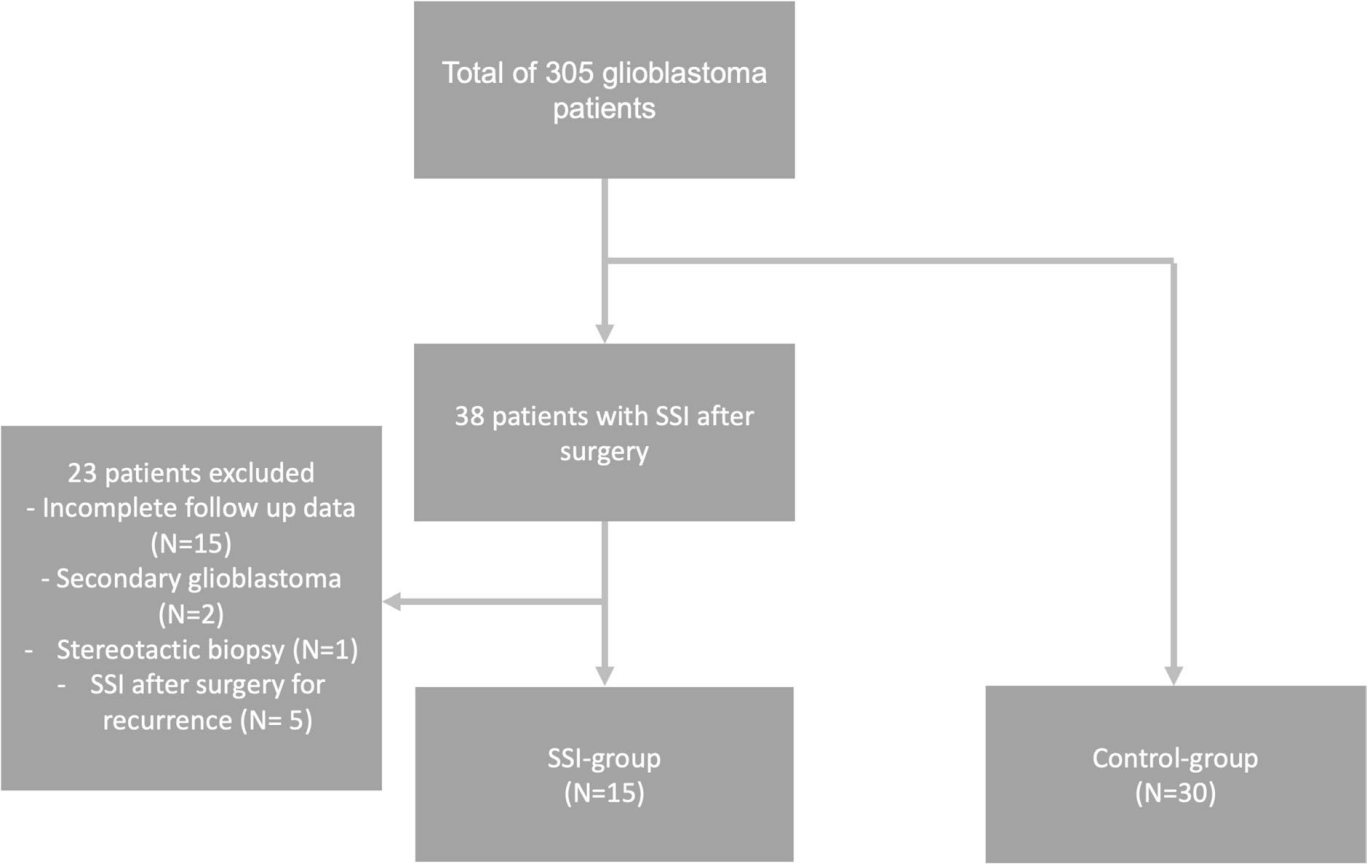
Flow chart. Out of 305 glioblastoma patients, 38 were identified to have SSI after surgery and 15 were included

A total of 305 patients underwent surgery for primary or recurrent glioblastoma in the Department of Neurosurgery at Hannover Medical School between 2010 and 2020. Of these, 38 patients were identified to suffer from SSIs after tumor resection. Two patients were lost to follow-up because they were living abroad. Thirteen patients had died but the exact date of death could not be ascertained. In two cases, glioblastoma was secondary to anaplastic astrocytoma (WHO grade 3). One patient had a stereotactic biopsy only. Five patients suffered from SSIs after surgery for recurrence. Those 23 patients were excluded, and the remaining 15 patients were included in the study (see Fig. 1).

The SSI group (N = 15) consisted of 5 men and 10 women while the control group (N = 30) included 18 men and 12 women (P = 0.37). Patients were aged between 9 and 70 in the SSI group and between 11 and 85 in the control group (SSI: 53.9 ± 4.9 years vs. control: 56.7 ± 3.5 years; P = 0.65). There was no significant difference in patients´ BMI between both groups (SSI: 28.49 ± 2.1 kg/m^2^ vs. controls: 26.17 ± 0.9 kg/m^2^; P = 0.55). The KPS at first diagnosis was 70 ± 6.1 in the SSI group, and 75 ± 2.3 in the control group (P = 0.42). Patients’ demographic data are summarized in Table 1.

**Table 1:** Patients’ demographic data

	SSI (N = 15)	Control (N = 30)	P-value
Age (years)	53.9 ± 4.9	56.7 ± 3.5	0.65
Sex			0.12
Men (%)	5 (33%)	18 (60%)	
Women (%)	10 (67%)	12 (40%)	
BMI (kg/m2)	28.49 ± 2.1	26.17 ± 0.9	0.55
Karnofsky Performance Score	70 ± 6.1	75 ± 2.3	0.42
Comorbidities			
Arterial hypertension (%)	6 (43%)	8 (57%)	0.5
Other malignancies (%)	2 (13%)	1 (3%)	0.25

Arterial hypertension was found in 6 patients in the SSI group and in 8 in the control group (P = 0.5). No patient suffered from diabetes mellitus. Two patients in the SSI group and one in the control group had other malignancies in their history (P = 0.25).

### Surgical site infections

Out of the 15 cases, there were 4 cases with superficial subcutaneous infection, 7 cases with epidural infection, and 4 cases with cerebritis or cerebral abscess. The bone flap was infected in 5 cases. Laboratory examinations at admission for SSI showed increased CRP in the most cases (mean: 47 ± 17 mg/L). The white blood count ranged from 4600 to 26,000 per microliter. Fourteen patients underwent surgical revision while one patient had conservative treatment with antibiotics only. The median time from glioblastoma resection to surgery for infection was 56 days. Microbiological examinations revealed Staphylococcus aureus in 5 cases followed by Cutibacterium acnes in 4 cases. Klebsiella pneumoniae and Staphylococcus epidermidis were found in 3 cases, respectively. All patients had antibiotic therapy; mainly cephalosporins and lincosamides.

### Glioblastoma course and treatment

All included patients underwent surgery for newly diagnosed glioblastoma. They had either gross total (SSI: N = 7, control: N = 16) or subtotal resection (SSI: N = 8, Control: N = 14; P = 0.75). Surgery was performed according to departmental standard techniques as described previously (Hong et al. 2013). Glioblastoma was diagnosed according to the “World Health Organization Histological Classification of Tumors of the Central Nervous System” (Louis et al. 2007, 2016). Isocitrate dehydrogenase (IDH) analysis revealed a mutation in 2 SSI patients and in 3 of the control cases (P > 0.99). Data are summarized in Table 2.

**Table 2:** Glioblastoma treatment

	SSI (N = 15)	Control (N = 30)	P-value
**IDH status** Wild type Mutated	13 (87%) 2 (13%)	27 (90%) 3 (10%)	> 0.99
**Extent of resection**			0.75
Gross total	7 (47%)	16 (53%)	
Subtotal	8 (53%)	14 (47%)	
**Time to adjuvant therapy (days)**	36.17 ± 4.7	30.31 ± 3	0.35
**Adjuvant therapy**			> 0.99
Yes	14 (93%)	28 (93%)	
No	1 (7%)	2 (7%)	

In the SSI-group, 10 patients had postoperative adjuvant radio-chemotherapy, 4 patients underwent radiotherapy only, and one patient had no postoperative adjuvant therapy. In the control group, 19 patients underwent postoperative radio-chemotherapy, 9 patients had radiotherapy only, and 2 patients had no postoperative treatment. The group comparison of the number of patients receiving an adjuvant therapy or not did not show a significant difference (P > 0.99). The time from surgery to the start of postoperative adjuvant therapy did not significantly differ between both groups (SSI: 36 ± 4.7 d vs. controls: 30.31 ± 3 d; P = 0.35).

**Fig. 2:**
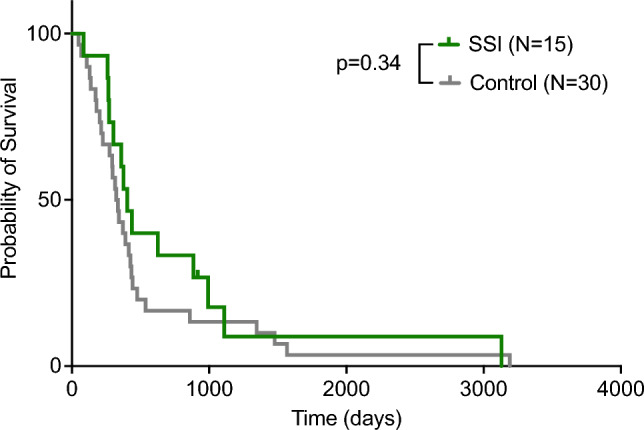
Kaplan–Meier curves estimating the overall survival in the SSI-group compared to the control group. Log-rank test and Gehan-Breslow-Wilcoxon test revealed no significant difference between both groups (*P* > 0.05)

**Fig. 3:**
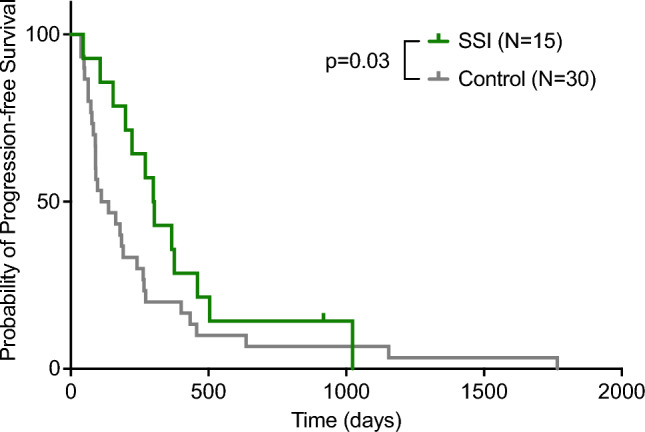
Kaplan–Meier curves of progression-free survival in both groups SSI group. Gehan-Breslow-Wilcoxon revealed a significantly higher median progression-free survival in the SSI group (*P* = 0.03)

The median overall survival was 403 days in the SSI group and 329 days in the control group (Log-rank: p = 0.34; Gehan-Breslow-Wilcoxon test: P = 0.19; see Table 3). Figure 2 shows the Kaplan–Meier curve estimating the overall survival in both groups. Subgroup comparisons based on age, gender and BMI did not reveal any differences. We specifically looked at the progression-free survival time in those patients with SSIs after primary surgery in order to evaluate the possible effects of SSI on the progress of glioblastoma. The median PFS was estimated to be 302 days in the SSI group and 124 days in the control group. The Kaplan–Meier estimation of progression-free survival is displayed in Fig. 3. While the curve comparison with the Log-rank test did not show a significant difference between both groups (P = 0.12), the Gehan-Breslow-Wilcoxon test yielded a significant disparity (P = 0.03). By the time we performed the analysis, one patient, who was still alive and had no recurrence, was censored at the day of analysis.

**Table 3:** Survival comparison

	SSI (N = 15)	Control (N = 30)	P-value
Median overall survival (days)	403 ± 12.9	329 ± 9.1	0.34
Progression-free survival (days)	302 ± 13.4	124 ± 9.1	0.03

**Fig. 4:**
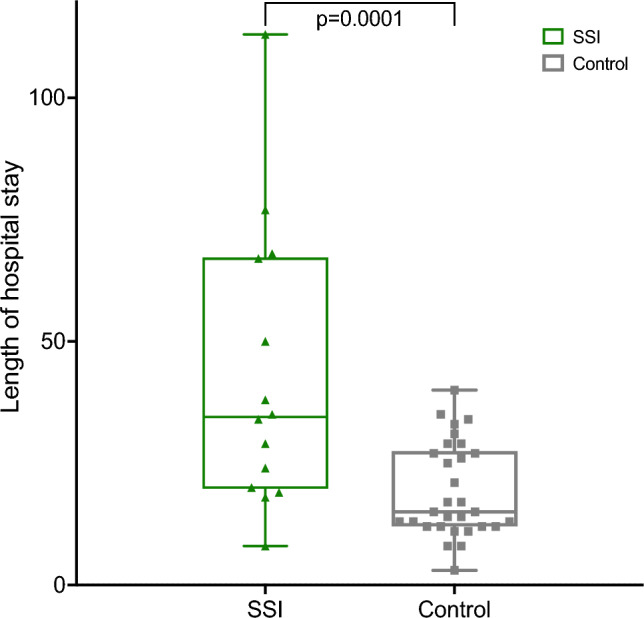
Box plot showing the distribution of LOS in the SSI group compared to the control group. LOS was significantly higher in the SSI group (***: *P* = 0.0001)

The number of hospitalizations was significantly higher in the SSI group (SSI: 3.2 ± 0.55 days; Control: 1.73 ± 0.13 days; P = 0.005; see also Table 4). Also, the total length of hospital stay was longer in the SSI group (SSI: 42.9 ± 7.7 days; control: 19.2 ± 1.8 days; P = 0.0001) (see Fig. 4).

**Table 4:** Hospitalization and length of stay

	SSI (N = 15)	Control (N = 30)	P-value
Number of hospitalizations	3.2 ± 0.55	1.73 ± 0.13	0.0005
Total length of stay (days)	42.9 ± 7.7	19.2 ± 1.8	0.0001

## Discussion

The potential effect of SSIs on survival has been a matter of investigation in different types of cancers. SSIs have mostly been reported to have either a negative or no effect on overall survival in other cancers (Atkinson et al. 2016; Lawler et al. 2020; Li et al. 2020; Yang et al. 2019). Also in glioblastoma, SSIs have been reported to have divergent effects on overall survival. A negative impact of SSIs on overall survival has recently been described in a multicentric study published by Salle et al. comparing the overall survival of 64 patients with SSI to an age-matched control group (Salle et al. 2021). Before that, available data suggested either a positive or no effect of SSIs on survival. An assumed survival benefit after SSI in glioblastoma was published in 1999 by Walker and Pamphlett concerning a 59-year-old woman who survived 5 years without recurrence after having SSI (Walker and Pamphlett 1999). In the same year, Bowles and Perkins reported another case of a 7-month-old infant who had a bone flap infection after glioblastoma surgery and survived for more than 10 years (Bowles and Perkins 1999).

Our SSI cohort contained one patient who survived for more than 8 years. Such isolated cases of unusually long survival after SSI in glioblastoma patients have been the subject of several publications (Bähr et al. 2009; Smoll et al. 2012). However, those reported long survival times might not be due to surgical site infections since they have also been often reported in patients without SSI. According to contemporary research, long term survival in glioblastoma is mainly related to the molecular biology of the tumor (Hertler et al. 2023). Tykocki and Eltayeb have systematically reviewed all reported cases of long survival in glioblastoma patients and listed the cases of 162 patients who survived longer than 10 years with no documented SSI (Tykocki and Eltayeb 2018). The longest reported survival time in their article was about 34 years.

A survival benefit of SSIs in glioblastoma patients has been described by De Bonis et al. in a case control study comparing 10 glioblastoma patients with SSI to an age-matched control-group (De Bonis et al. 2011). However, our results go against those findings, but they are in line with those of Chen et al. and Bohman et al., who also reported no effect of SSIs on overall survival time (Bohman et al. 2009; Chen et al. 2017). These divergent results might be due to the selected cohort, patients’ characteristics and most likely to possible interferences in the adjuvant radio-chemotherapy. In our study, SSIs did not lead to a significant delay of postoperative adjuvant radio-chemotherapy, which plays a crucial role in patients’ outcome.

Interestingly, in our study, progression-free survival was significantly higher in patients with SSI after primary surgery compared to the control group. The divergent results in the statistical tests (Log-rank vs. Gehan-Breslow-Wilcoxon) suggest that the potential effect of SSIs applies more to early recurrences and is limited in time. The hypothesized mechanisms of a potential survival benefit of SSI patients relate to local processes including a local immune reaction (competition between recruited immune cells and tumor cells for proteins and nutrients), activation of anti-tumor immune cells and release of specific cytokines or potential bacterial anti-cancer toxins (De Bonis et al. 2011; Hayes et al. 1995; Kazim et al. 2021; Löhr et al. 2013; Tanaka et al. 1980). Furthermore, it has recently been demonstrated, that bacterial peptides might activate tumor-infiltrating T-cells in glioblastoma and lead to an anti-tumor effect (Naghavian et al. 2023). In many patients, macroscopic infection signs were found without affecting the brain parenchyma. The above-mentioned mechanisms might not apply to those patients. The use of antibiotics and the associated gut dysbiosis might be another distinct pathway leading to changes in tumor growth in patients with SSI (Dono et al. 2022). Overall, the exact mechanisms leading to changes in PFS remain unclear. Further studies are needed to investigate those mechanisms.

In the second part of our study, we showed that SSIs led to more frequent hospitalizations and essentially doubled the LOS in glioblastoma patients. The reported increased length of stay is due to additional surgeries, need of intravenous antibiotics administration and decreased general condition. Although the association between SSIs and extended LOS is predictable and has been confirmed in other fields in adults and pediatric patients (de Lissovoy et al. 2009; Kirkland et al. 1999; Kusachi et al. 2012; Mahmoud et al. 2009; Monge Jodra et al. 2006; Sochet et al. 2017), it has not been quantified in glioblastoma patients yet. HRQOL in glioblastoma patients depends on various factors, including KPS, gender, tumor location and therapy regimen (Bergo et al. 2019; Coomans et al. 2022). Interestingly, hospitalizations have been shown to negatively affect patients´ HRQOL in diverse diseases including cancers (El-Jawahri et al. 2015; Jalal et al. 2022; Meira et al. 2015; Whitehouse et al. 2002). This likely also applies to glioblastoma.

One limitation of our study is the relatively small size of our cohort, especially due to the number of cases lost to follow-up, which did not allow subgroup comparison based on specific pathogens or infection localization. In our center, the O6-Methylguanine-DNA Methyltransferase (MGMT) methylation status has not been regularly determined in the past and therefore, was not available for inclusion in the statistical analysis for this study. Larger multicentric studies are needed to validate our findings. Furthermore, the retrospective design did not allow us to directly assess the effect of hospitalizations on the HRQOL by using a standardized questionnaire. Further studies are needed to quantify the effect of SSI on HQROL in glioblastoma patients.

In conclusion, we postulate that SSIs might reduce early recurrences without affecting overall survival. Furthermore, they might decrease HRQOL by doubling the total length of hospital stay.

## Supplementary Information

Below is the link to the electronic supplementary material.

Supplementary file1 (DOCX 18 KB)

## Data Availability

The datasets analyzed during the current study are available from the corresponding author on reasonable request.
