# Supplementary material for: Surgical site infections after glioblastoma surgery: boon or bane?
Source: J Cancer Res Clin Oncol. 2024 Jan 26;150(2):36. doi: 10.1007/s00432-023-05528-x (PMC10817840; doi:10.1007/s00432-023-05528-x)
Supplement: Supplementary file 1 — Supplementary file1 (DOCX 18 KB) [file 432_2023_5528_MOESM1_ESM.docx]

**Surgical site infections after glioblastoma surgery: boon or bane?**

**Supplementary data 1:** patients at risk for progression-free survival

| Time (days) | SSI (N=15) | Control (N=30) |
| --- | --- | --- |
| 0 | 15 | 30 |
| 37 |  | 30 |
| 45 | 14 |  |
| 48 |  | 28 |
| 50 |  | 27 |
| 63 |  | 26 |
| 73 |  | 24 |
| 77 |  | 23 |
| 82 |  | 22 |
| 89 |  | 21 |
| 90 |  | 20 |
| 91 |  | 18 |
| 97 |  | 17 |
| 107 | 13 |  |
| 111 |  | 16 |
| 137 |  | 15 |
| 154 | 12 |  |
| 163 |  | 14 |
| 179 |  | 13 |
| 184 |  | 12 |
| 190 |  | 11 |
| 199 | 11 |  |
| 222 | 10 |  |
| 240 |  | 10 |
| 263 |  | 9 |
| 266 |  | 8 |
| 271 | 9 |  |
| 272 |  | 7 |
| 300 | 8 |  |
| 303 | 7 |  |
| 366 | 6 |  |
| 376 | 5 |  |
| 401 |  | 6 |
| 433 |  | 5 |
| 457 |  | 4 |
| 460 | 4 |  |
| 504 | 3 |  |
| 636 |  | 3 |
| 917 | 2 |  |
| 1023 | 1 |  |
| 1154 |  | 2 |
| 1766 |  | 1 |

**Supplementary data 2:** patients at risk for overall survival

| Time (days) | SSI (N=15) | Control (N=30) |
| --- | --- | --- |
| 0 | 15 | 30 |
| 49 |  | 30 |
| 68 |  | 29 |
| 84 | 15 |  |
| 108 |  | 28 |
| 129 |  | 27 |
| 137 |  | 26 |
| 172 |  | 25 |
| 179 |  | 24 |
| 202 |  | 23 |
| 213 |  | 22 |
| 225 |  | 21 |
| 259 | 14 |  |
| 266 | 13 |  |
| 271 | 12 |  |
| 274 |  | 20 |
| 293 |  | 19 |
| 296 |  | 18 |
| 303 | 11 |  |
| 314 |  | 17 |
| 322 |  | 16 |
| 335 |  | 15 |
| 342 |  | 14 |
| 359 | 10 |  |
| 372 |  | 13 |
| 376 | 9 |  |
| 390 |  | 12 |
| 403 | 8 |  |
| 414 |  | 11 |
| 426 |  | 10 |
| 434 |  | 9 |
| 438 | 7 |  |
| 442 |  | 8 |
| 476 |  | 7 |
| 536 |  | 6 |
| 625 | 6 |  |
| 859 |  | 5 |
| 885 | 5 |  |
| 917 | 4 |  |
| 992 | 3 |  |
| 1110 | 2 |  |
| 1346 |  | 4 |
| 1477 |  | 3 |
| 1567 |  | 2 |
| 3130 | 1 |  |
| 3190 |  | 1 |
